# A New Fossil of Necrotauliidae (Insecta: Trichoptera) from the Jiulongshan Formation of China and Its Taxonomic Significance

**DOI:** 10.1371/journal.pone.0114968

**Published:** 2014-12-10

**Authors:** Yujia Liu, Weiting Zhang, Yunzhi Yao, Dong Ren

**Affiliations:** 1 Key Lab of Insect Evolution and Environmental Changes, Capital Normal University, Beijing, 100048, China; 2 Geoscience Museum, Shijiazhuang University of Economics, Shijiazhuang, 050031, China; The National Orchid Conservation Center of China; The Orchid Conservation & Research Center of Shenzhen, China

## Abstract

**Background:**

*Acisarcuatus variradius* gen. et sp. nov., an extinct new species representing a new genus, is described from the Middle Jurassic Jiulongshan Formation in Daohugou Village, Inner Mongolia, China.

**Methodology/Principal Findings:**

In this paper, we revised the diagnosis of Necrotauliidae Handlirsch, 1906. One new genus and species of Necrotauliidae is described. An analysis based on the fossil morphological characters clarified the taxonomic status of the new taxa.

**Conclusions/Significance:**

New fossil evidence supports the viewpoint that the family Necrotauliidae belongs to the Integripalpia.

## Introduction

The Amphiesmenoptera, comprising two distinctive insect orders: the Trichoptera and the Lepidoptera [1]. Trichoptera, or caddisflies, are holometabolous insects. Their bodies and wings are covered by bushy hairs, and the adults resemble moths in appearance. They are among the largest group of aquatic insects [1], [2] and one of the most diverse groups of insects overall with more than 14,000 extant species and more than 680 fossil species [3]. Trichoptera include three living suborders: Annulipalpia, Integripalpia, and Spicipalpia [4], but the monophyly of Spicipalpia is disputable [2], [5]. Species of the Permian suborder Protomeropina Sukatcheva (1980) [6] are sometimes placed in Trichoptera [7], [8] and sometimes are considered representatives of the Amphiesmenoptera stem group or more distant lineages [9], [10].

The Necrotauliidae Handlirsch, 1906, an extinct caddisflies family, has been considered as representatives of the Amphiesmenoptera stem-group [1], [11]–[16]. Since the original description definition imprecise, at one time the family was deemed to “primitive” Trichoptera-like Mesozoic insects [12], [17]–[21]. However, the stem-group of Trichoptera is exactly similar to that of Lepidoptera. This ambiguity has augmented the heterogeneity of the Necrotauliidae [22]. Necrotauliidae have been described in the Late Triassic of Western Europe and the Late Mesozoic of Asia. Most fossil specimens of Necrotauliidae collected from Germany, Russia, China, and United Kingdom [13], [14], [16], [23], [24]. In China four Mesozoic Necrotauliidae, including *Necropsis paludis* Hong, 1983 *Necrotaulius fascialatus* Hong, 1983, *N. kritus* Lin, 1986, and *N. qingshilaense* Hong, 1984 have been described [13], [25].

Here, we describe a new and unique male adult fossil specimen collected from the Daohugou beds. The beds consist of 100–150 m thick succession of grey-white or locally reddish, thinly bedded claystones, shales, siltstones and sandy mudstones with intercalated ash-fall tuffs and ignimbrites. It was radiometrically dated by ^40^K/^40^Ar at 164–165 Ma [26], which accorded with the Callovian–Oxfordian boundary interval of the latest Middle Jurassic, using the latest standard international time scale [27]. Although there has been controversial to the precise age and stratigraphic position [28], [29]. The well-preserved fossils of insects and other animals also prove that the Daohugou fauna assemblages may correlate with the Middle Jurassic Yan-Liao biota [30]. This new fossil specimen is significant because of its well-preserved head, maxillary palps, fore- and hind wings, abdomen, and male genitalia. Most previously described representatives of the family were based only on fragmentary remnants and/or isolated wings [23], [31]–[33]. Thus, this new fossil is an important supplement to Necrotauliidae records and provides new evidence for studying their origin and evolution. The complete preservation of the new specimen enables us to determine the phylogenetic status of Necrotauliidae.

## Materials and Methods

### Material

The part and counterpart of the fossil specimen (CNU-TRI-NN2013001pc) are deposited in the Key Lab of Insect Evolution & Environmental Changes, College of Life Sciences, Capital Normal University, Beijing, China. No specific permits were required for the described field studies.

### Nomenclatural Acts

The electronic version of this document does not represent a published work according to the International Code of Zoological Nomenclature (ICZN), and hence the nomenclatural acts contained in the electronic version are not available under that Code from the electronic edition. Therefore, a separate edition of this document was produced to ensure that numerous identical and durable copies were simultaneously obtainable (from the publication date noted on the first page of this article) to provide a public and permanent scientific record in accordance with Article 8.1 of the Code.

This published work and the nomenclatural acts it contains have been registered in Zoobank, the online registration system for the ICZN. The Zoobank LSIDs (Life Science Identifiers) can be resolved and the associated information viewed through any standard web browser by appending the LSID to the prefix “http://zoobank.org”. The ISID for this publication is: urn:lsid:zoobank.org:pub:64E83F92-0277-4F41-BC5B-9732B6691611. The electronic edition of this work was published in a journal with an ISSN, and has been archived and is available from the following digital repositories: PubMed Central and LOCKSS.

### Methods

The fossil specimen was examined using a Leica MZ12.5 dissecting microscope (Wetzlar, Germany) and illustrated with the aid of a drawing tube attachment. When observing the details, the specimen was put under pure alcohol. Line drawings were made by Photoshop 9.0 graphic software (Adobe Systems, San Jose, CA, USA). Photographs were taken with a Nikon Digital Camera DXM 1200C (Tokyo, Japan).

Body length was measured from the apex of the head to the apex of the abdomen. The wing length was measured from the base to the apex of the wing. The length of antennae was measured from the base to the apex.

Interpretation and terminology used herein follow Holzenthal et al. [5]: C, costa; Sc, subcosta; R, radius; R_1_, branches of anterior radius; Rs, posterior branch of radius (composed of R_2_, R_3_, R_4_, and R_5_); M, media; M_1+2_, anterior branch of media, composed of M_1_ and M_2_; M_3+4_, posterior branch of media, composed of M_3_ and M_4_; Cu, cubitus; Cu_1_, anterior branch of cubitus (composed of Cu_1a_ and Cu_1b_); Cu_2_, posterior branch of cubitus; 1A, 2A, and 3A, first, second, and third branches of anal vein; the forks giving rise to R_2_ and R_3_, R_4_ and R_5_, M_1_ and M_2_, M_3_ and M_4_, CuA_1a_ and CuA_1b_, are referred to as F1, F2, F3, F4, and F5, respectively; the discoidal cell (Dc) is the cell formed by the branching of Rs into R_2+3_ and R_4+5_ and is closed apically by the sectorial crossvein (s); the medial cell (Mc) is formed by the branching of M into M_1+2_ and M_3+4_; the anal cells delimited by 1A, 2A, and 3A.

## Results

### Systematic Paleontology

Family Necrotauliidae Handlirsch, 1906.

#### Type genus

*Necrotaulius* Handlirsch, 1906.

#### Revised diagnosis

Head with setal warts. Antennae filiform. Maxillary palps five-segmented, segment V terminal, invisibly annulated, not covered densely hair or scales. Pronotum with one pair of setal warts. Mesothorax with triquetrous scutellum. Tibial spur formula: 0: 2: 3 or 4?. Forewing with vein Sc long, extending into pterostigma region; pterostigma variously developed; Rs with 4 branches; M usually with 4, rarely 3 branches, crossveins weakly developed or absent. Hind wing, Sc long; Rs with 4 branches; M with 3 or 4 branches; anal veins not joined.

#### Remark

According to the new fossil, we added the characters on head, antennae, maxillary palps and tibial spur formula. On hind wing, M with only 3 branches on the previously reported specimens, but our specimen has M four-branched, thus we revised this character.

Genera included. *Acisarcuatus* gen. nov.; *Cretotaulius* Sukacheva, 1982 [19]; *Karatauliodes* Sukacheva, 1968 [34]; *Karataulius* Sukacheva, 1968 [34]; *Mesotrichopteridium* Handlirsch, 1906 [12]; *Necropsis* Hong, 1983 [35]; *Necrotaulius* Handlirsch, 1906 [12]; *Pteromixanum* Sukatcheva and Jarzembowski, 2001 [23]; *Scyphindusia* Sukacheva, 1985 [36].

*Acisarcuatus* gen. nov.

urn:lsid:zoobank.org:act:85FB6752-52E4-4BFE-8549-9D43976642BB.

#### Type Species

*Acisarcuatus variradius* gen. et sp. nov.

#### Diagnosis

Warts present on head. Forewing with 5 forks (I–V); Sc forked, Sc_2_ straight and long, extending into pterostigma region; anal veins form a typical anal loop; discoidal cell short and closed, median cell and thyridial cell very long and open. Male genitalia harpagones regularly curving mesad, narrowing at apex; median phallic apparatus seems to be spicular and arcuate.

#### Etymology

The generic name is a combination of the Latin word *acis* (tip) and *arcuatus* (arc, curve), describing the peculiar curving of R1; gender masculine.

#### Distribution

China.

#### Remark

We assigned *Acisarcuatus variradius* gen. et sp. nov. to the Necrotauliidae on the basis of the following characters: head with anterior and posterior setal warts; maxillary palps five-segmented, first segment stout; tibial spur formula: 0: 2: 3 or 4?; forewing Sc long, extending into pterostigma region; Rs with 4 branches; M with 4 branches; crossvein rare; hind wing Sc extending about 2/3 length of hind wing, Rs and M with 4 branches; anal veins not joined; male genitalia inferior appendages two-segmented gonopods, with dense hairs around margin.

This new specimen shows affinity on vein characters with some other genera of Necrotauliidae. *Acisarcuatus* share several characters with *Necrotaulius* Handlirsch, 1906 [12] such as warts on the head are present, forks I–V long and slender, and crossvein m-cu_1_ present. However, *Acisarcuatus* differs from *Necrotaulius* in: 1) Sc forked (vs. Sc unforked); 2) R_1_ unforked, straight in basal part but curved in pterostigma area (vs. R_1_ forked and straight in pterostigma area); 3) Dc short and closed by r_3_–r_4_ (vs. Dc open); 4) Rs_1+2_ furcation before Rs_1+2_ furcation (vs. Rs_1+2_ furcation beyond Rs_1+2_ furcation).

*Acisarcuatus* differs from *Mesotrichopteridium* Handlirsch, 1906 [12] in the following characters: 1) forewing length 0.9 mm (vs. forewing length 3.5–4.5 mm); 2) forewing without crossvein sc-r (vs. crossvein sc-r present); 3) M four-branched on the hind wing, (vs. M_4_ reduced).

*Acisarcuatus* differs from *Pteromixanum* Sukatcheva and Jarzembowski, 2001 [23] in the following characters: 1) body size relatively large, length 0.9 mm (vs. length 0.5 mm); 2) Sc forked (vs. Sc unforked); 3) forewing with forks I–V (vs. forewing with forks I, II, III, V); 4) M forking before Rs forking (vs. M forking beyond Rs forking).

**Figure 1:**
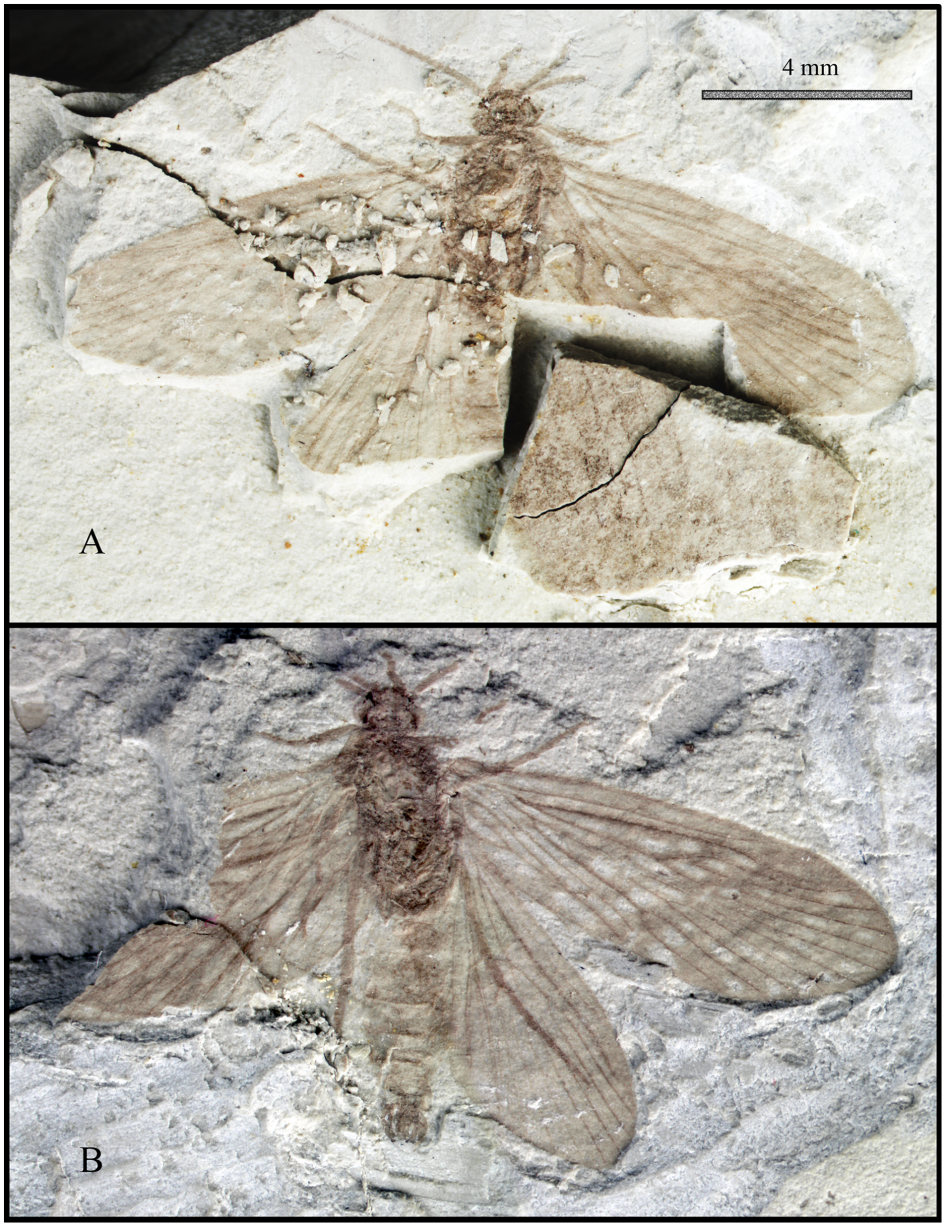
Photographs of the holotype of *Acisarcuatus variradius* gen. et sp. nov. CNU-TRI-NN2013001pc. **A,** ventral view, **B,** dorsal view.

*Acisarcuatus variradius* **gen. et sp. nov. (** **Figs. 1** **–** **3** **)**.

**Figure 2:**
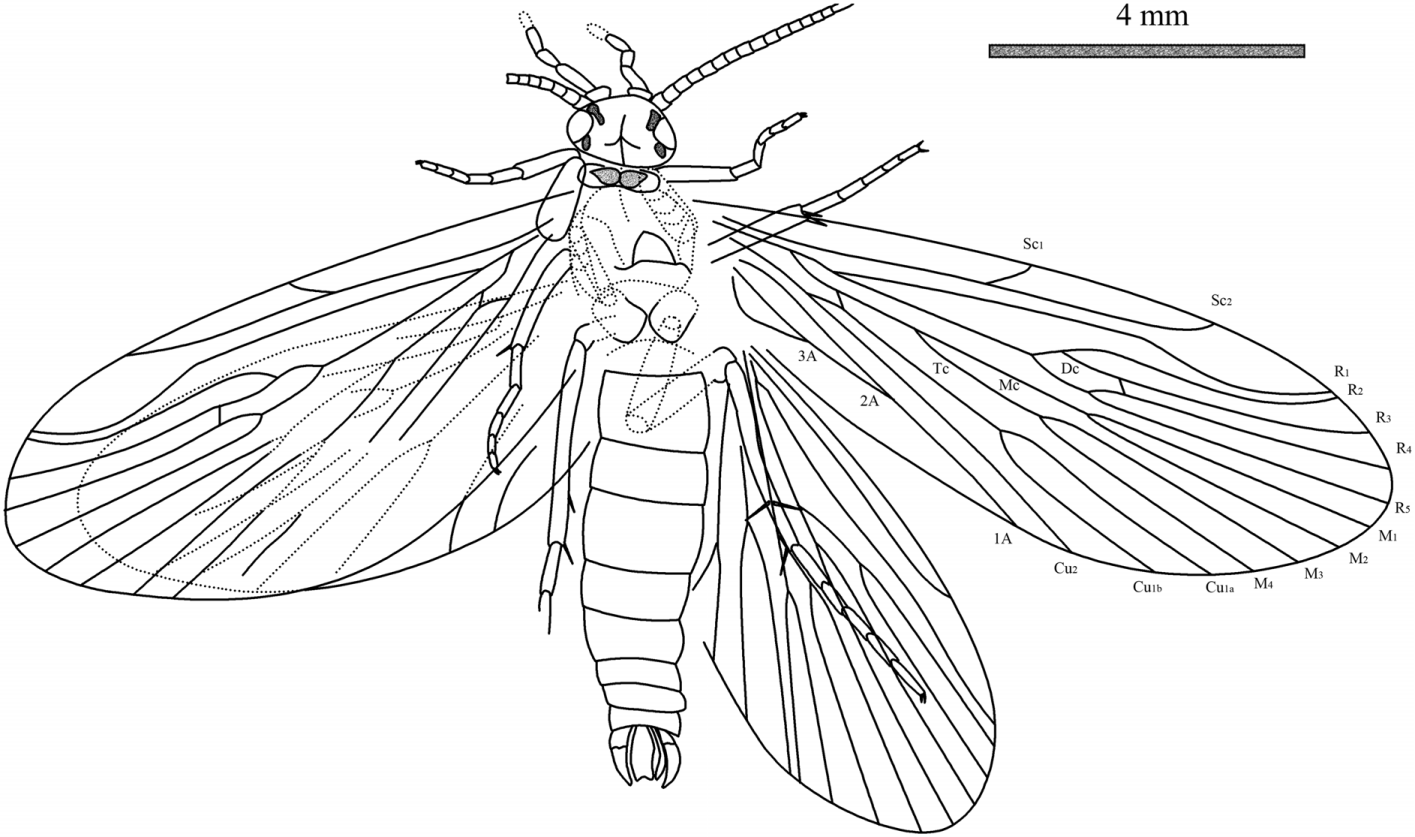
Line drawings of *Acisarcuatus variradius* gen. et sp. nov. CNU-TRI-NN2013001pc.

**Figure 3:**
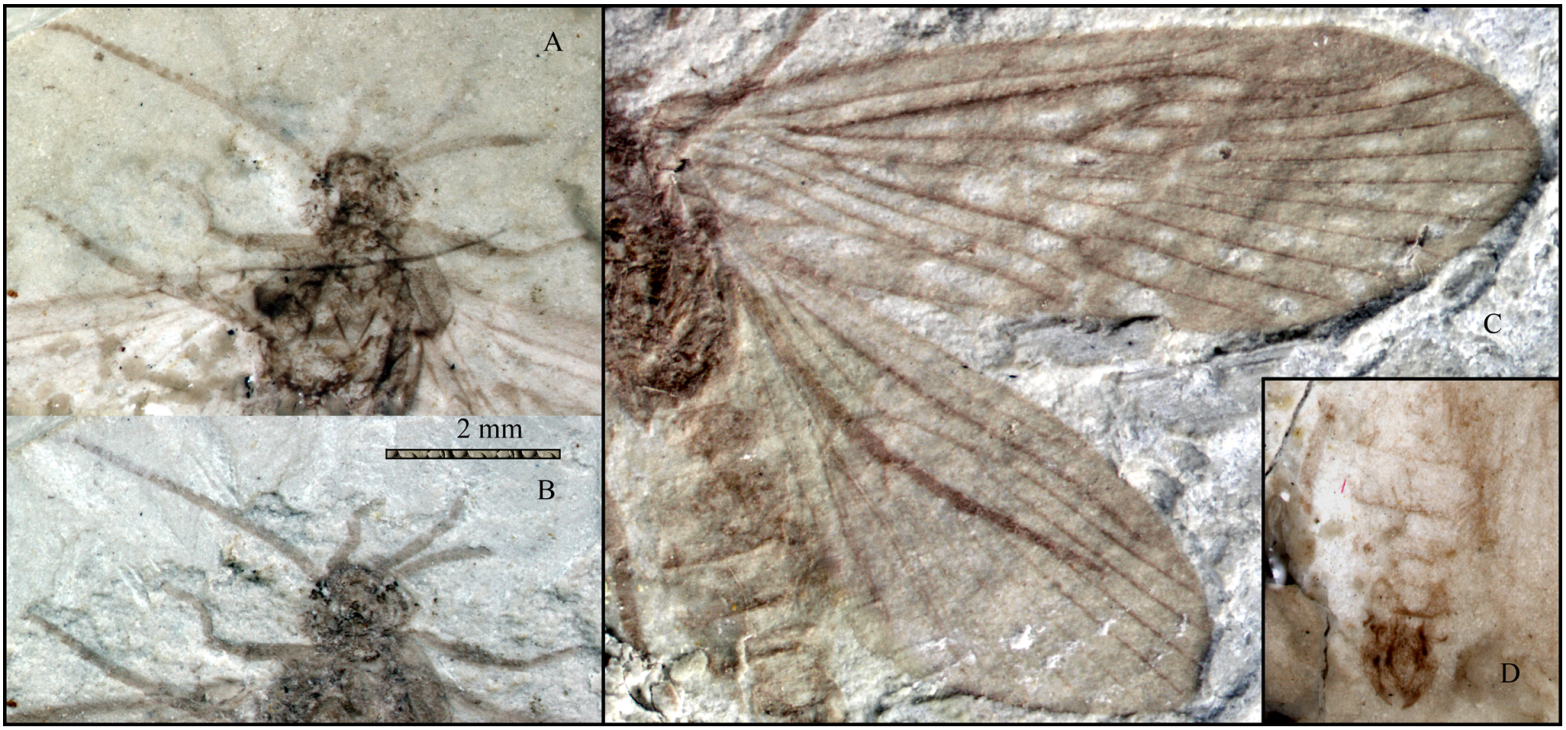
Photographs of *Acisarcuatus variradius* gen. et sp. nov. CNU-TRI-NN2013001pc. **A,** head, antennae, maxillary palps, fore leg, and mid leg in alcohol. **B,** head, antennae, maxillary palps, fore leg, and mid leg. **C,** forewing and hind wing. **D,** male genitalia in alcohol.

urn:lsid:zoobank.org:act:D1107F5B-EAB9-4C76-B0F2-89CF7264A77F.

#### Type Material

Holotype, male: CNU-TRI-NN2013001pc (part and counterpart, dorsoventrally compressed). Antennae, maxillary palps, setal warts on head and thorax, tibial spurs, forewing, hind wing and male genitalia are well-preserved.

#### Locality and horizon

Daohugou Village, Shantou Township, Ningcheng County, Inner Mongolia, China (N41°18.979′, E119°14.318′), Jiulongshan Formation, Middle Jurassic.

Etymology. Specific name is a combination of the Latin word *vari* (different) and *radius*, indicating peculiar R_1_; gender masculine.

#### Diagnosis

Body small; Sc 2-branched; R_1_ unforked, straight basally and curved in pterostigma area, R_1_ closed to Rs_1_ terminally.

#### Description

Head with saponaceous triangle, compound eye at head sides, oval. Anterior setal warts and posterior setal warts present surrounding compound eye, irregularly oval. Antennae filiform but not well-preserved, scape slightly thicker than pedicel and flagellum, pedicel cylindrical, flagellum slender, length of segments equal to their diameter. Maxillary palps five-segmented; segment I swollen, segment II longest, segment III subequal to IV, segment V indistinct.

Thorax: Pronotum with one pair of setal warts, symmetrically drop-shaped. Mesothorax with triquetrous scutellum. Legs well-preserved. Foretibial spur invisible, mesotibia with two apical spurs; metatibia with two preapical spurs and one or two apical spurs; tibial spur formula: 0: 2: 3 or 4. Fore tarsus five-segmented, slender, segment I longest, II-IV subequal in length; mesotarsus five-segmented, all tarsal segments with terminal spinules. Tarsal claw visible. Forewings elongated elliptic; Rs_4_ terminating slightly below apex of forewing. Forewing with forks I–V; Sc forked, Sc_2_ slightly bend terminally and ending into C at about 2/3 the length of forewing, Sc_1_ terminating into C at about 2/3 length of Sc; R_1_ unforked distally, straight in basal part and curved in pterostigma area; Rs forked at mid-length of the forewing; Dc short and closed by r_3_–r_4_; F1 forks before than F2; Rs_1_ slight bent towards R_1_ at terminus; M originating from base of R; M forking before Rs forks; F3 and F4 longer than their stems; F3 forks later than F4; Mc very long and apparently open; Cu_1_ bifurcated into Cu_1a_ and Cu_1b_, and then F5 forks as same level as Rs fork; crossvein m-cu_1_ present; Tc open; Cu_2_ straight and simple; anal veins visible, 1A straight, 2A reaches the median of 1A, 3A strongly curved and reaches median of 2A. Hind wing narrower and shorter than forewing. Hind wing with forks I–V; Dc, Mc, and Tc open; Sc simple; R_1_ straight and simple; F1 forks later than F2, F3 forks slightly before F4, F5 forks earliest.

Abdomen: In dorsal view, eight sternites visible and male genitalia prominent, bearing pair of two-segmented gonopods; coxopodite broad at base and shorter than harpago. Harpagones regularly curving mesad, narrowing at apex. Coxopodites and harpagos with dense hairs around margin. Middle preanal appendage and periphallus visible, median phallic apparatus seems to be spicular and arcuate. External structural details of male genitalia indistinct in fossil.

#### Remark

In our specimen, only one apical spur is visible, but the presence of another apical spur cannot be excluded (i.e. absent due to incomplete preservation).

#### Measurements (in mm)

Body length 9.92, width 1.74. Head length 0.91, width 1.43. Interocular space 0.75. Maxillary palp segments I–IV: 0.15, 0.52, 0.30, 0.30. Scutellum length 0.42, width 0.57. Forewing length 9.36, width 3.40. Hind wing length 6.79, width 3.02. Fore leg length: femur 1.09, tibia 0.79, tarsus I–V: 0.38, 0.30, 0.23, 0.23, 0.23; middle leg length: tarsus I–V: 0.52, 0.34, 0.30, 0.26, 0.26; hind leg length: tibia 2.38, tarsus I–V: 0.64, 0.42, 0.42, 0.42, 0.49.

## Discussion

Kristensen provided a summary of 21 apomorphies supporting the monophyletic group of Amphiesmenoptera, with both Trichoptera and Lepidoptera certain features (e.g. forewing the terminal of the anal vein fusion) [37]. Furthermore, the monophylies of Trichoptera and Lepidoptera are also generally accepted [9]. Insect fossil caddisflies are generally preserved incompletely or indistinctly, and often only forewing is visible on the fossil [32], [33]. Many paleontologists considered Necrotauliidae to be a representative of the amphiesmenopteran stem-group, and proximal to the common ancestor of trichopterans and lepidopterans that survived after the Triassic [1], [11]–[14], [17]–[21], [38]. This viewpoint is mainly based on the characteristics of forewing.

Our specimen possesses very clear male genitalia with harpagones (coxopodite broad at base and shorter than harpago), middle preanal appendage, spicular and arcuate median phallic apparatus. The harpagones is a synapomorphy of Trichoptera [5], [24]. Beside that, maxillary palps of the new fossil specimen correspond to the apomorphy of Integripalpia [39], [40]: maxillary palps upturned, with segment I swollen, densely hairs or scales invisible, segment II longest, segment III subequal to IV. The character that crossvein m absent is also similar to suborder Integripalpia [40]. On the basis of these characters, we believe Necrotauliidae is belongs to Integripalpia (Trichoptera).

Meanwhile *A. variradius* gen. et sp. nov. has some plesiomorphies of Integripalpia: Sc forked; forewing with five forks; crossveins very rare on both forewing and hind wing; only two crossveins, r and m-cu_1_ present. These characters also can be found in the extinct suborder Protomeropina [6], [41]–[43]. It is interesting to speculate that necrotauliids are representatives of the Integripalpia stem-group rather than the amphiesmenopteran.
